# Heterogeneous lupus‐specific lesions and treatment outcome, in a single patient, over a period of time

**DOI:** 10.1002/ccr3.2105

**Published:** 2019-03-22

**Authors:** Melissa Fernandes, Anna V. Taulaigo, Carolina Vidal, Patrick Agostini, Nuno Riso, Maria Francisca Moraes‐Fontes

**Affiliations:** ^1^ Unidade de Doenças Auto-imunes/Serviço de Medicina 7.2, Hospital de Curry Cabral Centro Hospitalar Universitário de Lisboa Central Lisboa Portugal; ^2^ Serviço de Medicina Interna Hospital do Divino Espírito Santo Ponta Delgada, Açores Portugal; ^3^ Laboratório de Anatomia Patológica Centro Hospitalar Universitário do Algarve Faro‐Portimão Portugal

**Keywords:** discoid lupus erythematosus, heterogeneity, lupus erythematosus tumidus, subacute cutaneous lupus erythematosus, systemic lupus erythematosus, treatment

## Abstract

The report highlights the importance of strict clinico‐histological correlations when skin biopsies are performed in diagnostic doubt in systemic lupus erythematosus. Furthermore, PUVA is never indicated in autoimmune conditions involving photosensitivity, due to high potential for internal and cutaneous aggravation of the disease, as the authors observed in this case.

## 1 INTRODUCTION

Systemic lupus erythematosus (SLE) remains a disease of unknown etiology with a mosaic of clinical presentations. Cutaneous lesions are a first sign of SLE in up to one quarter of patients.^1^ According to histopathologic criteria, cutaneous manifestations include lupus erythematosus (LE)‐specific and LE‐nonspecific lesions. LE‐specific lesions are subdivided according to clinical phenotype, histological changes, laboratory abnormalities, and average duration. As the clinical and histological features of SLE skin lesions may mimic many other dermatological conditions, a skin biopsy may be required and a correct diagnosis relies on strict clinico‐pathological correlation, benefiting from evaluation by a lupus expert or an experienced dermatopathologist.^1^, ^2^, ^3^ We hereby describe diagnostic and management difficulties and a successful therapeutic outcome in a single SLE patient, applying current knowledge to discuss a multiplicity of cutaneous lesions.

## 2 CASE REPORT

**Figure 1:**
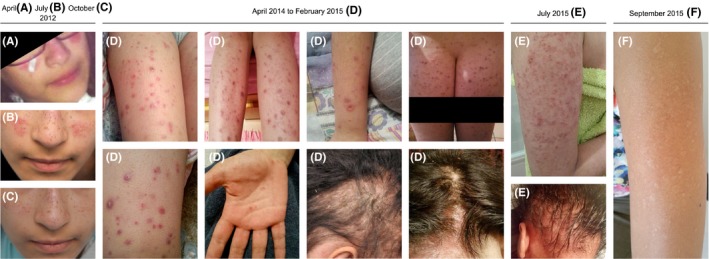
Clinical features. Age 12—cutaneous lesions localized to malar regions (A and B); topical treatment led to improvement without scarring (C); Age 14—generalized rash, started in legs and extending to arms, buttocks, palm of hands and fronto‐temporal regions of the scalp with alopecia (D); Age 15—post PUVA (E); Age 15—post rituximab (F)

**Figure 2:**
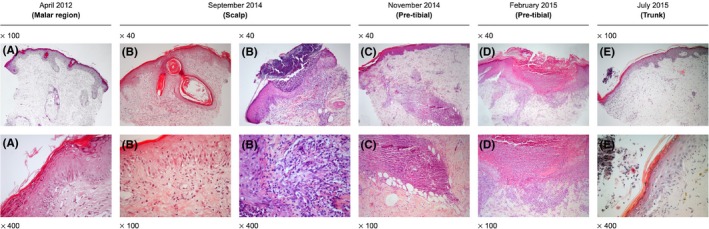
Histological features. Temporal correlation with photographs in Figure 1 per lesion: Age 12—localized to malar regions (A); Age 14—scalp (B), pre‐tibial (C); Age 15—pre‐tibial, pre PUVA (D); Age 15—trunk, post PUVA (E)

**Figure 3:**
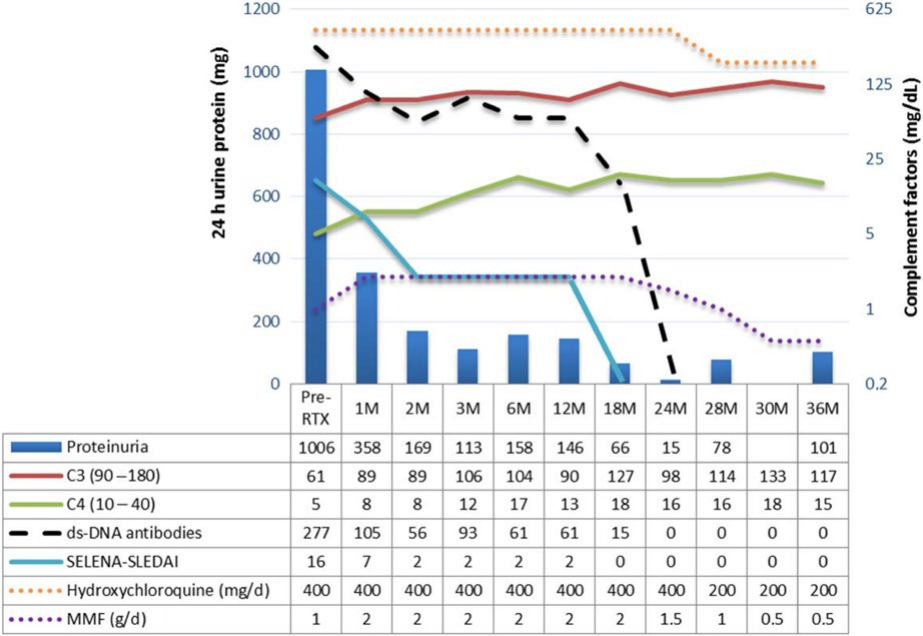
Follow‐up disease activity  measured by the safety of estrogens in lupus national assessment‐systemic lupus erythematosus disease activity index (SELENA‐SLEDAI) and therapy.

In April 2012, a previously healthy 12‐year‐old female presented with a malar rash (Figure 1A). Menarche had started at 11 years of age, and the patient had been vaccinated according to the national Portuguese vaccination program including the first dose of the human papilloma virus vaccine, administered 1 month before symptom onset. The clinical characteristics, histological reports, treatments, and outcome are presented in chronological order in Tables 1 and 2. A skin biopsy (Figure 2A) was reported as compatible with a diagnosis of lupus. More specifically, there was a thin epidermis, the basement membrane was not thickened, and a mild perivascular lymphocytic infiltrate and focal vacuolization were found at the dermoepidermal junction. Edema, vessel ectasia, a mild perivascular lymphocytic infiltrate, and mucin deposits were found in the reticular dermis and a lymphocytic infiltrate surrounded hair follicles. At that time anti‐SSA antibodies were present, but there were no other abnormalities in the full blood count, renal function, or urinary sediment. There was improvement with topical hydrocortisone, tacrolimus, and photoprotection. One month later, the patient developed fever and lost 1.5 kg in weight, and 3 months later, the rash on the cheeks returned (Figure 1B). Repeat biopsies in the malar region were performed in July 2012 but a tissue orientation error prevented interpretation. At that time, a lupus band test from unaffected skin revealed the presence of IgM and IgG granular deposits in the basement membrane. Hydroxychloroquine (HCQ) 400 mg/d was started and the rash improved (Figure 1C). Despite HCQ, in December 2012, symmetrical painful violaceous lesions appeared on the tip of the fingers and toes. These resolved with deflazacort 30 mg/d for 1 week, progressively discontinued in the following 3 months. In June 2013, still on HCQ, worsening of the malar rash was documented. In April 2014, the patient reported the onset of pruritic well‐defined hyperkeratotic papules initially in the lower limbs, rapidly spreading to the buttocks, upper torso, arms, palms of hands and scalp, resulting in severe alopecia (Figure 1D). The complete full blood count, hepatic and renal function tests were within normal ranges. A more extensive profile revealed ANA positivity (1/1280), with an elevated anti‐dsDNA, a low C4 and C3. The patient was then treated with daily deflazacort 30 mg, azathioprine (AZA) 50 mg and anti‐histaminics, with no improvement. At that time, scabies was suspected and topical treatment with benzyl benzoate was prescribed on two occasions. Several scalp punch biopsies in September 2014 (Figure 2B) were reported as compatible with lupus, folliculitis being reported in one of the samples (Figure 2C). No periodic acid‐Schiff (PAS) positive microorganisms were identified, and there was no immunoglobulin deposition by direct immunofluorescence. The skin condition progressively deteriorated, and both deflazacort and AZA were discontinued. Several discordant histological diagnosis of perforating dermatosis (Figure 2D) and psoriasis (Figure 2E) ensued. The patient was then treated with oral isotretinoin, whole body psoralen, and ultraviolet‐A light therapy (PUVA), 3 times a week (oral 8‐Methoxsalen administered before each session with initial, final and total doses of 1.5, 9, and 29.5 J/cm^2^, respectively). These treatments were harmful and stopped after eleven sessions due to the development of generalized, erosive, painful and extremely pruritic disseminated cutaneous lesions with severe alopecia (Figure 1E), after which the patient was admitted to our unit in July 2015. Laboratory tests showed leucopenia (3100/μL), neutropenia (1680/μL), ANA positivity (1/640), anti‐dsDNA antibodies (277 IU/mL; ELISA reference: <25 IU/mL), complement consumption (C3 = 61 mg/dL [normal range: 90‐180 mg/dL], C4 = 5 mg/dL [normal range: 10‐40 mg/dL]), and sustained proteinuria (highest value: 1006 mg/24 h). ELISA tests for anti‐Beta‐2 Glycoprotein1 and anti‐cardiolipin antibodies as well as the lupus anticoagulant assay were negative. The renal biopsy revealed class V membranous glomerulonephritis with granular deposits of immunoglobulins, complement components, and light chains (Figure S1); tissue and serum anti‐Phospholipase A2 receptor antibody were negative. In view of her skin condition, off‐label intravenous immunoglobulin (IVIG) was administered (20 g/d × 5 days) together with HCQ 400 mg/d, and mycophenolate mofetil (MMF) was started at the dose of 500 mg bd and increased weekly by 250 mg bd to a maximum dose of 1 g bd, together with enalapril 5 mg/d. On the 20th day of hospitalization due to the ongoing severity of the skin lesions, the patient was treated with rituximab (RTX) 1 g preceded by methylprednisolone 500 mg, on days 1 and 15, in addition to the above‐mentioned drugs. The skin rash resolved within 2 weeks of the RTX administration, with residual hypopigmentation (Figure 1C); full hair re‐growth was documented at 6 months (Figure 1D) with well‐being and sustained renal remission at 3 years of follow‐up, allowing for successful medication taper (Figure 3), continuing HCQ and MMF as maintenance treatment.

**Table 1:** Clinical characteristics and sequential histological reports

	Date (Age, y)	April 2012 (12)	July 2012 (12)	December 2012 (12)	April 2014 (14)	September 2014 (14)	November 2014 (14)	February 2015 (15)	July 2015 (15)
Rash	Location	Malar region, nasal bridge (Figure 1A)	Malar region (Figure 1B)	Fingers and toes	Legs, buttocks, arms, trunk, palms of hands and scalp, sparing the face (Figure 1D)				Entire integument involved, severe alopecia and malar rash (Figure 1E)
	Description	Symmetrical scaly erythematous, hyperkeratotic plaques	Symmetrical erythematous, edematous plaques, smooth surface	Symmetrical painful violaceous lesions	Extremely pruritic well‐defined papulosquamous psoriasiform lesions. Lesions in lower limbs described as “folliculitis‐like.” Scaly erythematous lesions in fronto‐parietal scalp regions with alopecia				Symmetrical generalized erythematous excoriated lesions and malar rash
Histology report	Punch biopsy site	Malar region (Figure 2A)	Tissue orientation error	—	—	Scalp (Figure 2B)	Pre‐tibial (Figure 2C)	Pre‐tibial Figure 2D)	Trunk (Figure 2E)
	Epidermis, papillary dermis	Thin epidermis, basement membrane not thickened. Mild perivascular lymphocytic infiltrate	No interpretation	—	—	Epidermal acanthosis, orthokeratosis and folicular plugging. In one of the biopsies there is acute suppurative folliculitis with no microorganisms (PAS negative)	Thin epidermis	Normal epidermis, erosion with pustule	Irregular epidermal acanthosis, subcorneal pustules and intracorneal microabscesses
	Dermoepidermal junction	Focal vacuolization		—	—	Vacuolar interface	No change Direct IF negative	—	—
	Reticular dermis	Edema, vessel ectasia. Mild perivascular lymphocytic infiltrate. Mucin deposits		—	—	Edematous with lymphoplasmacytoid infiltrate. Fibrinoid and mucin deposits	Lymphoplasmacytoid infiltrate.	—	Discrete perivascular lymphocytic infiltrate
	Pilo‐sebaceous units and eccrine glands	Lymphocytic infiltrate surrounding hair follicles		—	—	Fibrosis of hair follicles	Intense lymphoplasmacytoid infiltrate surrounding hair follicles. PAS negative	—	—
Additional information		*Clinical information*: Lupus *Histological diagnosis*: Discoid lupus	*Clinical information*: Lupus *Histological diagnosis*: none		Some lesions ulcerated. Scabies suspected	*Clinical information*: Lupus *Histological diagnosis*: Lupus	*Clinical information*: Scabies? Pityriasis lichenoides chronica? *Histological diagnosis*: Folicullitis	*Clinical information*: Lymphomatoid papulosis? *Histological diagnosis*: reactive perforating collagenosis vs perforating folliculitis	*Clinical information*: Lupus, lymphoma? *Histological diagnosis*: psoriasis

**Table 2:** Laboratory results, treatments and outcomes

	April 2012	July 2012	December 2012	April 2014	September 2014	November 2014	February 2015	July 2015
LABORATORY TESTS	Anti‐nuclear antibody (ANA) positive, anti‐SSA Ab positive (double‐ID assay). Anti‐CdsDNA negative. No other changes.	ANA positive, anti‐SSA Ab positive (method unavailable)	None	ANA 1/1280 (IF – speckled), anti‐dsDNA 163 [(N < 20 IU/mL ‐ RIA), anti‐Sm, anti‐SSA present (double‐ID assay)), C4 4 (N 10‐40 mg/dL); C3 63 (N 90 – 180 mg/dL)	ANA positive; anti‐dsDNA 110 (N < 20 IU/mL ‐ RIA), C4 6 (N 10‐40 mg/dL); C3 63 (N 90 – 180 mg/dL	None	None	ANA positive (1/640), anti‐dsDNA 277 (N < 100 IU/mL ‐ ELISA, C4 5 (N 10‐40 mg/dL); C3 61 (N 90 – 180 mg/dL Sustained proteinuria (highest value: 1006 mg/24 h) Class V membranous glomerulonephritis with granular deposits of immunoglobulins, complement components and light chains
TREATMENT	Topical hydrocortisone and tacrolimus	Started HCQ 400 mg/day	Maintained HCQ DFZ 30 mg/day for one week – ↓ over 3 months	Maintained HCQ Re‐started DFZ 30 mg/day Azathioprine 50 mg/day Hydroxyzine 25 mg tds. Two treatments with topical benzyl benzoate.	Maintained HCQ DFZ 30 mg/day Azathioprine 50 mg/day.	All treatment was stopped	Oral isotretinoin Whole body psoralen and ultraviolet‐A light therapy, 3‐times a week (oral 8‐Methoxsalen administered before each session with initial, final and total doses of 1,5 J/cm2, 9 J/cm2 and 29,5 J/cm2, respectively).	IVIG 20 g/day x 5 days Restarted HCQ Micophenolate mofetil Enalapril 20th day hospitalization: Riruximab 1 g preceeded by Methylprednisolone 500 mg, repeated two weeks later
OUTCOME	Improvement without scarring	Improvement without scarring (Fig. 1 c)	Resolution without scarring	No improvement. New lesions continued to appear.	No improvement. New lesions continued to appear.	No improvement. New lesions continued to appear.	All treatments were stopped after nine sessions (March 25 to April 20) due to the development of generalized, painful and pruritic crusts with aggravated alopecia, malar rash, fever and generalized lymphadenopathy	Complete healing with areas of depigmentation in arms (Fig. 1f) Disease remission (Figure 3)

Ab: antibody; DFZ: Deflazacort; ID: immunodiffusion; HCQ: Hydroxychloroquine; IF: immunofluorescence; RIA: radioimmunoassay.

## 3 DISCUSSION AND CONCLUSIONS

In contrast to lupus nephritis where a renal biopsy has prognostic and therapeutic value with a classification based on well‐recognized features,^4^ when lupus affects the skin, lesions cannot be distinguished on the grounds of histology alone.^1^ Classically, in most cases of SLE, mucin deposition in the dermis is reportedly prominent. Findings may be subtle, with discrete basal cell liquefactive degeneration, papillary dermal edema and perivascular and perifollicular mild chronic inflammatory infiltrate, indistinguishable from subacute cutaneous lupus erythematosus (SCLE) and discoid lupus erythematosus (DLE).^5^, ^6^ There are, however, histopathological features that are more frequent in some cutaneous subtypes.^7^ We envisage the following scenario based on a retrospective clinico‐pathological correlation: In April 2012, at disease onset, the patient may have presented with acute cutaneous lupus erythematosus (ACLE), suggested by a scaly localized malar rash. Nevertheless, this was somewhat atypical for ACLE, as the rash was very discrete, there were no systemic features and the lack of scarring after healing was against a diagnosis of DLE. The findings of perivascular and periadnexal lymphocytic infiltration together with mucin deposits, no epidermal change and no thickened basement membrane, were in favor of lupus erythematosus tumidus (LET). The latter corresponded to the morphology of the lesions, characterized by symmetrical erythematous and edematous plaques with a smooth surface and no scales, registered 3 months later, in July 2012. Although HCQ was started, the patient presented 8 months later with a rash on the tip of the fingers and toes suggestive of chilblain lupus. Almost 1 year later, in June 2013, the skin lesions worsened on the face, possibly due to ACLE or LET, as there was no residual scarring. From April 2014, we believe the patient presented with SCLE and SLE, on the basis of papulosquamous lesions that spared the central face and laboratory findings. These lesions were highly pruritic, psoriasiform, and not exclusive to sun‐exposed areas. By September 2014, histological findings (orthokeratosis and follicular plugs) were suggestive of scalp DLE leading to intensification of immunosuppression. From then on, a combination of atypical features (the highly pruritic and psoriasiform nature of the lesions), misleading clinical information and refractoriness to therapy distanced the diagnostic path away from SLE, the underlying disease. Several misdiagnosis including scabies, folliculitis, a histological diagnosis of reactive perforating collagenosis vs perforating folliculitis and even psoriasis were evoked at the time, leading to an incorrect treatment choice with PUVA, with severe deleterious consequences. At the time of PUVA treatments, we propose the patient was affected by SCLE, with generalized skin lesions on the entire integument, in addition to SLE. Complete healing with no alopecia and no scarring contradict the diagnosis of scalp DLE. Finally, the hypopigmentation that remained after healing was typical for photosensitive SCLE. In summary, the patient seems to have developed several lupus‐specific skin lesions over time, starting at least 2 years before the criteria for the diagnosis of SLE were fulfilled.^8^, ^9^ Different manifestations appeared over time. Initially, ACLE/LET responding favorably to HCQ, immunosuppressants, and sunscreen, and subsequently, SCLE, refractory to therapy. Contrarily to its reportedly favorable prognosis,^10^ LET seems to have preceded SLE in this patient. Of note, PUVA treatment is a formal contraindication in patients with photosensitivity.

Metabolic disorders and chronic pruritis may be associated with reactive perforating dermatosis. This is a variant of prurigo nodularis, histologically characterized by epidermal perforation^11^ for which ultraviolet (UV) light therapy is recommended.^12^ But there was no evidence of epidermal perforation and not unexpectedly, in this patient, UV light therapy was equivalent to a major form of photoprovocation, with a deleterious effect, aggravating pre‐existing and precipitating new cutaneous lesions, followed by a renal flare. Furthermore, lesions affecting the palms would not be expected to occur in any type of folliculitis. The use of IVIG was justified by the severity of the presentation. The positive long‐term response to rituximab with a steroid sparing effect has been previously described,^13^, ^14^ contrasting with the adverse events associated to the prolonged use of systemic steroids in juvenile SLE patients with skin involvement.^15^, ^16^

This report emphasizes the divergence of cutaneous lupus manifestations that may present in a single patient over a period of time and the importance of clinico‐pathological correlation for a correct diagnostic and therapeutic approach.

## CONSENT

The Subject and her Mother have given informed consent for publication.

## CONFLICT OF INTEREST

None declared.

## AUTHOR CONTRIBUTION

MF and AVT: share co‐first authorship in drafting the manuscript. MF, AVT, CV, NR, and MFMF: responsible for acquisition, analysis, and interpretation of data; NR and MFMF: overall responsibility for patient care and critical manuscript review.
