# Supplementary figures and images for: Heterogeneous lupus‐specific lesions and treatment outcome, in a single patient, over a period of time
Source: Clin Case Rep. 2019 Mar 22;7(5):865–71. doi: 10.1002/ccr3.2105 (PMC6509922; doi:10.1002/ccr3.2105)

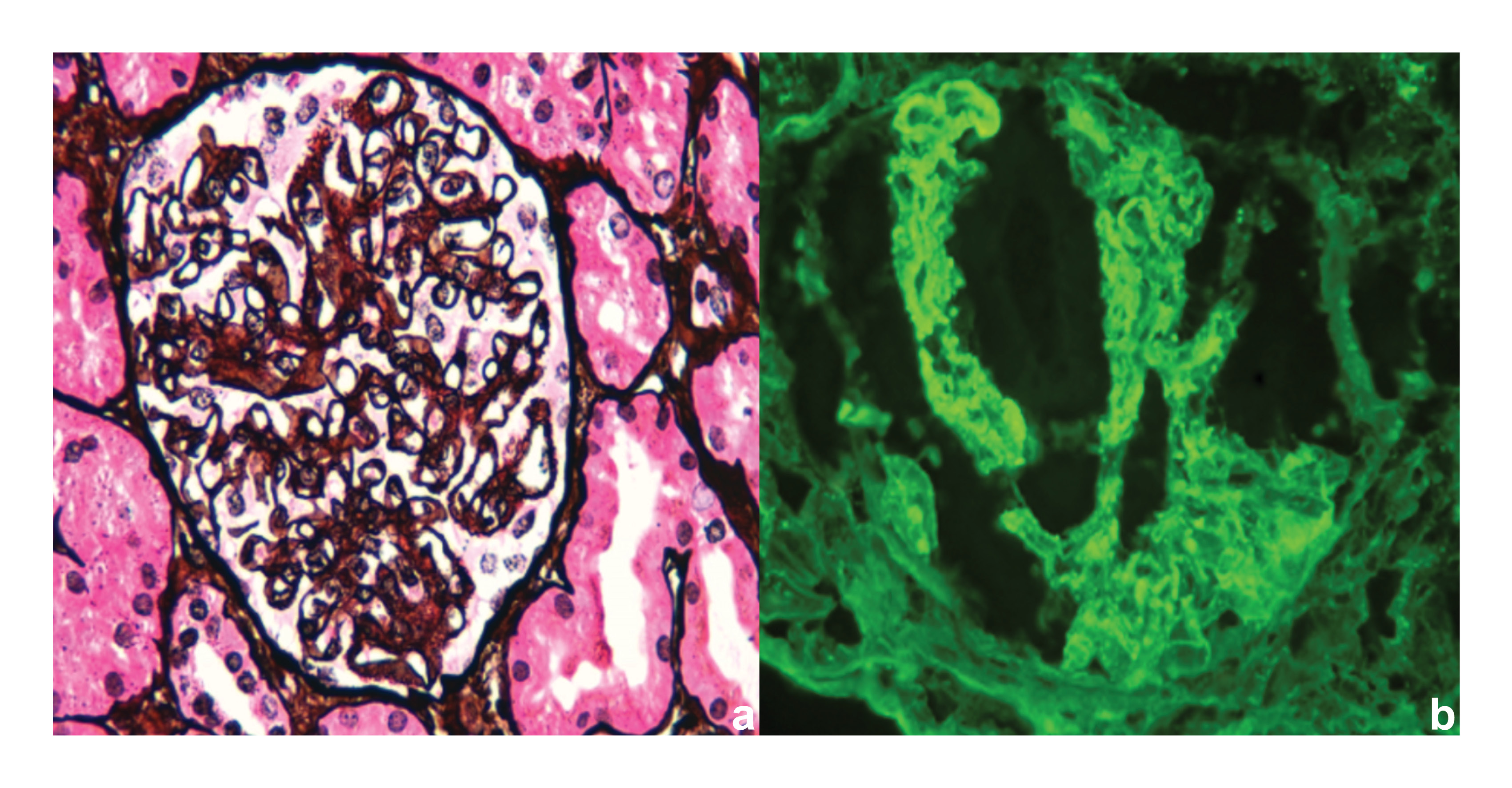

Supplement: Supplementary file 1 [file CCR3-7-865-s001.jpg]
